# Management of Non-Islet Cell Tumor Hypoglycemia Induced by Adrenal Cortical Carcinoma

**DOI:** 10.1210/jcemcr/luaf245

**Published:** 2025-11-05

**Authors:** Yaling Tang, Oksana Hamidi, Jessica Abramowitz, Azucena Herrera Chancay, Liwei Jia, Sadia Ali

**Affiliations:** Department of Internal Medicine, University of Texas Southwestern Medical Center, Dallas, TX 75390, USA; Division of Endocrinology and Metabolism, Department of Internal Medicine, University of Texas Southwestern Medical Center, Dallas, TX 75390, USA; Division of Endocrinology and Metabolism, Department of Internal Medicine, University of Texas Southwestern Medical Center, Dallas, TX 75390, USA; Division of Endocrinology and Metabolism, Department of Internal Medicine, University of Texas Southwestern Medical Center, Dallas, TX 75390, USA; Department of Pathology, University of Texas Southwestern Medical Center, Dallas, TX 75390, USA; Division of Endocrinology and Metabolism, Department of Internal Medicine, University of Texas Southwestern Medical Center, Dallas, TX 75390, USA

**Keywords:** non-islet cell tumor hypoglycemia, adrenal cortical carcinoma, Cushing syndrome, insulin-like growth factor 2

## Abstract

Non-islet cell tumor hypoglycemia (NICTH) is a rare paraneoplastic syndrome that significantly worsens the prognosis of underlying malignancies. It is characterized by hypoglycemia resulting from ectopic production of insulin-like growth factor 2. NICTH caused by adrenal cortical carcinoma (ACC) is extremely rare. To date, no curative treatment of nonoperable NICTH has been reported. In this report, we describe a case of ACC-induced NICTH that was refractory to multiple treatments for hypoglycemia. It provides diagnostic and treatment approaches for NICTH and emphasizes the limitations of existing medical therapies for NICTH.

## Introduction

Non-islet cell tumor hypoglycemia (NICTH) is a rare and debilitating condition, which significantly worsens the prognosis of underlying tumors. Its incidence rate is estimated as one per million person-years [1]. The most common underlying malignancy of NICTH is fibrous tumors, whereas adrenal cortical carcinoma (ACC) is extremely rare as a cause [1, 2]. The mechanism of hypoglycemia for NICTH is through excess production of insulin-like growth factor 2 (IGF-2) and IGF-2 precursors secreted by underlying tumors and its insulin-like downstream effects mediated via insulin receptor activation. Tumors overexpress IGF-2 ribonucleic acid (RNA) and produce large pre-pro-IGF-2 molecules. These molecules bind with IGF-binding protein 3 (IGFBP3) to form binary complexes, whereas normal IGF-2 molecules circulate as a ternary complex after binding with IGFBP3 and acid-labile subunit, creating a higher ratio of binary to tertiary complexes in NICTH than in the physiologic state. These binary complexes easily cross epithelial barriers and binds to insulin receptors. Furthermore, free IGF-2 is often elevated, which inhibits insulin, growth hormone (GH) and its downstream IGF-1 levels. Consequently, insulin and IGF-1 are typically low during the profound hypoglycemia episodes [3]. Although IGF-2 precursors are often elevated, the level of total IGF-2 can be either normal or elevated in these cases. Therefore, elevated IGF-2 to IGF-1 ratio is used to diagnose NICTH, of which the consensus cutoff is 10:1 [4]. To date, the only curative treatment for NICTH is complete tumor removal. However, many tumors are not amenable to complete surgical resection and do not respond completely to other anticancer therapies. While novel anti-hypoglycemic therapies are used in the clinical setting, evidence of their impact on NICTH is limited.

Here we describe a challenging case of a patient with ACC-induced NICTH, which was refractory to multiple standard and investigational treatments. This case is unique as NICTH developed in recurrent/metastatic ACC, and it promoted exploration of the current data on investigational treatments for NICTH.

## Case Presentation

**Figure 1.**
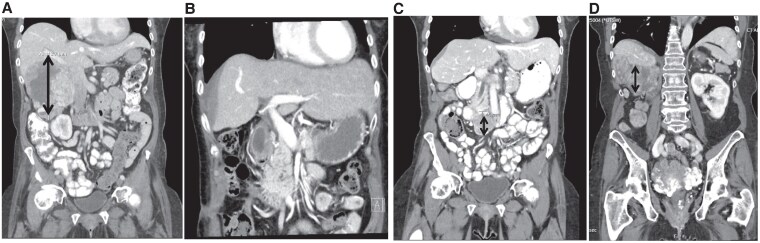
Computed tomography of the abdomen and pelvis showing the adrenal mass across the clinical course. (A) At the initial diagnosis; (B) After right adrenalectomy, nephrectomy, and partial hepatectomy; (C) At the detection of recurrent aortocaval node; (D) At the onset of non-islet cell tumor hypoglycemia. The double-sided arrow indicates the location of the adrenal mass at each time point.

**Figure 2.**
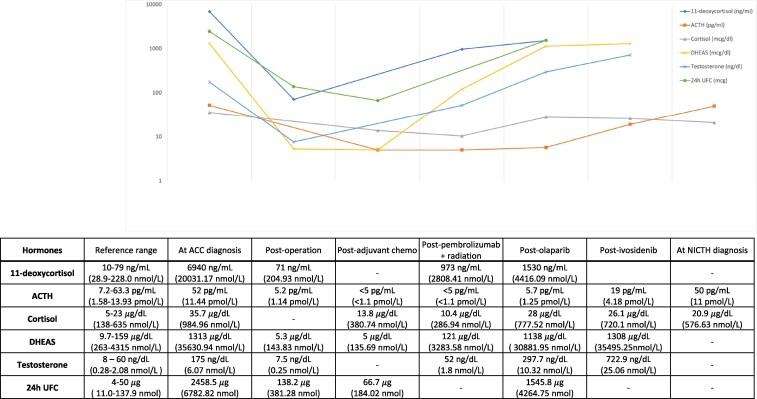
Adrenal hormonal studies across clinical course. Abbreviations: ACC, adrenal cortical carcinoma; ACTH, adrenocorticotropic hormone; DHEAS, dehydroepiandrosterone sulfate; NICTH, non-islet cell tumor hypoglycemia; UFC, urine free cortisol.

**Figure 3.**
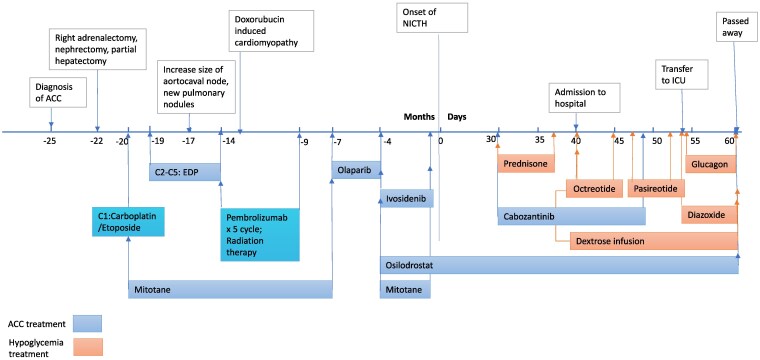
Treatment approaches applied across clinical course. EDP is cisplatin, etoposide, doxorubicin. Olaparib is a PARP (poly-polymerase) inhibitor, which blocks DNA repair; it was applied due to tumor V1446fs*42 mutations. Ivosidenib is an IDH1 (isocitrate dehydrogenase-1)inhibitor; it was applied due to tumor IDH1 R132H mutations. Osilodrostat inhibits aldosterone synthase and 11beta-hydroxylase, thus inhibits cortisol and aldosterone synthesis. Cabozantinib, a small tyrosine kinase inhibitor, is a new approved ACC medication. Abbreviations: ACC, adrenal cortical carcinoma; ACTH, adrenocorticotropic hormone; C, cycle; ICU, intensive care unit; NICTH, non-islet cell tumor hypoglycemia.

A 62-year-old woman presented with severe hypoglycemia after 2 years of ACC diagnosis. At the initial diagnosis of ACC, she presented with new-onset hypertension, diabetes, skin darkening, lower extremity edema, unintentional weight loss, and generalized weakness. Computed tomography (CT) of the abdomen and pelvis showed a large 13-cm heterogenous right adrenal mass invading the inferior vena cava and abutting the liver and duodenum (Fig. 1). Biochemical workup showed elevated cortisol, testosterone, 11-deoxycortisol, and dehydroepiandrosterone sulfate (DHEA-S) (Fig. 2). Plasma metanephrine and normetanephrine were normal. She underwent tumor resection and pathology revealed high grade, Stage pT3N1 ACC (ENSAT stage III) with extensive tumor necrosis, mitotic rate of 24 per 50 high power field, and Ki-67 of 20% to 30%. The tumor was 15.8 cm × 11.2 cm × 9.7 cm in size, and its surgical resection margin was negative. The adrenocorticotropic hormone (ACTH) staining of the tumor was negative. After surgery, adrenal hormone excess improved (Fig. 2). She was treated with adjuvant mitotane and chemotherapy with cisplatin, etoposide, and doxorubicin (40 mg/m^2^ for cycle 2-3, and 38 mg/m^2^ for cycle 4-5; 1.79 m^2^, total dose 260 mg) (Fig. 3). The treatment was complicated with doxorubicin-induced cardiomyopathy with a left ventricle ejection fraction (LVEF) of 43% and grade 1 diastolic dysfunction. On 6-month postoperative surveillance imaging, she was found to have local retroperitoneal nodal tumor recurrence and lung metastasis (Fig. 1). She received pembrolizumab for 5 cycles and consolidative radiation therapy, but additional lymph node metastases were detected (Fig. 3). She then received targeted therapy for somatic mutations *ATM* V1446fs*42 and *IDH1* R132H with olaparib for 15 weeks and ivosidenib for 18 weeks, respectively, but did not derive clinical benefit (Fig. 3). Upon progression, she began experiencing episodes of diaphoresis and shortness of breath, and laboratory analysis revealed new-onset symptomatic hypoglycemia.

## Diagnostic Assessment

An extensive hypoglycemic workup showed serum glucose 46 mg/dL (2.56 mmol/L), undetectable insulin < 1.0 μIU/mL (< 6 pmol/L), C-peptide 1.7 ng/mL I (0.57 nmol/L), beta-hydroxybutyrate 0.1 mmol/L (within normal range), proinsulin 4.5 pmol/L, IGF-2 > 1315 ng/mL (> 172 nmol/L), IGF-I 34 ng/mL (4.46 nmol/L), and significantly elevated IGF-2 to IGF-1 ratio (> 38). Morning serum cortisol was 20.9 µg/dL (576.63 nmol/L) and ACTH was 50 pg/mL (11 pmol/L), excluding adrenal insufficiency (Table 1).

**Table 1.** Hypoglycemia workup at the onset of non-islet cell tumor hypoglycemia

Test	Results	Reference
Blood glucose	38 mg/dL (2.1 mmol/L)	—
Hemoglobin A1c	5.50%	—
Beta-hydroxybutyrate	0.1 mmol/L	0.0-0.3 mmol/L
Insulin	< 1.0 μIU/mL (< 6.0 pmol/L)	2.6—2.49 μIU/mL (18—179 pmol/L)
C-peptide	1.7 ng/mL (0.57 nmol/L)	1.1—4.4 ng/mL (0.37 -1.46 nmol/L)
Cortisol	20.9 µg/dL (577 nmol/L)	5—23 µg/dL (138—635 nmol/L)
ACTH	50 pg/mL (11.0 pmol/L)	7.2—63.3 pg/mL (1.58—13.93 pmol/L)
IGF-1	34 ng/mL (4.46 nmol/L)	34 -194 ng/mL (4.46 -25.45 nmol/L)
IGF-2	> 1315 ng/mL (> 172 nmol/L)	—
IGF-2 to IGF-1 ratio	> 38	—

In cases of NICTH, the expected laboratory findings typically include: insulin < 3 μIU/mL (18 pmol/L), C-peptide < 0.3 nmol/L (0.9 ng/mL), IGF-1 < 100 ng/mL (13 nmol/L), IGF-2 > 275 ng/mL (36.67 nmol/L), and an IGF-2 to IGF-1 ratio > 3:1—often exceeding 10:1 in confirmed cases. ACTH and cortisol levels are typically within normal range or elevated, aiding to exclude adrenal insufficiency.

Abbreviations: ACTH, adrenocorticotropic hormone; IGF, insulin-like growth factor; NICTH, non-islet cell tumor hypoglycemia.

## Treatment

A 1-week trial of prednisone 30 mg daily was initiated outpatient. This did not improve hypoglycemia and was discontinued. The patient was admitted to the hospital for management of ongoing episodes of severe hypoglycemia and was started on intravenous (IV) dextrose. She was started on a 1-week trial of octreotide 100 mcg 3 times daily and later given 1 dose of pasireotide 40 mg, both of which were not successful. She was subsequently treated with diazoxide at 5 mg/kg/day, divided by every 8 hours, but hypoglycemic episodes continued to increase in both frequency and severity. She was transferred to the intensive care unit for closer observation and started on IV glucagon infusion. IV dextrose and glucagon infusion were titrated and dextrose concentration was increased from D15 to D30 to D50 and glucagon from 0.2 mg/h to 0.3 mg/h. Glucose infusion rate varied throughout the hospital stay based on volume status, tube feed rate, kidney function, and diuretics timing and dosage. She also received an additional dose of pasireotide 20 mg subcutaneously (total dosage 60 mg/month). Cabozantinib was added to treat ACC for 18 days but was discontinued due to pancytopenia (Fig. 3).

## Outcome and Follow-Up

The hospital course was complicated by volume overload despite aggressive diuresis which limited the administration of IV treatments. She developed decompensated heart failure and kidney failure. Her clinical condition deteriorated with a progressive decline in urine output, and respiratory and mental status. Due to the overall poor prognosis and declining clinical status, she was transitioned to comfort care and died 2 months after the NICTH diagnosis.

## Discussion

We report a rare case of ACC-induced NICTH that was refractory to multiple hypoglycemia treatments. Approximately 80% of ACCs overexpress IGF-2 [5]. Yet, aberrant secretion of IGF-2 causing paraneoplastic hypoglycemia is uncommon in the setting of ACC. A systemic review of IGF-2-mediated NICTH included 233 patients and found fibrous tumors to be the most common underlying malignancy (53.2%). Only 8 prior cases of adrenal tumors (3.4%) were previously reported with IGF-2 mediated hypoglycemia [1]. Of the available literature with detailed clinical course, the presentation of hormone excess, such as cortisol, was not clinically evident; rather, all patients presented with constitutional symptoms such as weight loss and anorexia [6-10]. IGF-2 levels were variable, either within the normal range, slightly reduced, or elevated, whereas IGF-2 to IGF-1 ratio was significantly elevated (> 10:1) in all cases for diagnosis of NICTH. Three cases had unsuppressed ACTH [6, 8, 9] and in one of these, concomitant ACTH secretion was confirmed by immunohistochemical staining [6]. Our case showed no clinical or radiologic evidence of hypothalamic, pituitary, or ectopic ACTH production, despite unsuppressed ACTH level and negative ACTH staining in the ACC. All nonoperable cases eventually developed metastases, had poor response to chemotherapy, and either died [6-8, 10] or were lost to follow-up [9].

Treatment of NICTH can be divided into 2 approaches: (i) definitive treatment of underlying malignancy; and (ii) acute management of hypoglycemia, used independently or combined. The only curative treatment for stage I-III ACC is surgical resection, but many ACCs at the time of diagnosis are unresectable or metastatic, and they recur after surgery. Our patient received multiple treatments for ACC including surgery, radiation, immunotherapy with pembrolizumab, cabozantinib, and other investigational therapies including olaparib, and ivosidenib with limited clinical benefit.

Regarding hypoglycemia, IV dextrose can help treat low glucose values in the hospital setting but does not address the underlying cause and therefore is not able to prevent further episodes of hypoglycemia. Prior reports suggest the modest effectiveness of recombinant human GH, glucocorticoids, and continuous glucagon infusion in hypoglycemia management from NICTH [3]. In our case, we did not attempt GH due to presence of metastatic disease. Glucocorticoids were ineffective and glucagon merely allowed for a brief reduction of the IV dextrose rate. IV infusion rates were eventually limited by decompensated heart failure and renal failure. The trials of diazoxide, pasireotide, and octreotide were also unsuccessful [3].

Our group has previously reported the successful use of pasireotide for the treatment of hypoglycemia in a case of NICTH caused by hepatocellular carcinoma [11]. The mechanism was thought to be the interaction of pasireotide with a high proportion of somatostatin receptor type 5 (SSTR5) expressed in hepatocellular carcinoma, leading to the lowering of IGF-2 levels. Available data show the staining intensity of SSTR5 in ACC of approximately 14% to 24% [12, 13], which likely explains the lack of response to pasireotide in our case.

Alpelisib is a promising emerging medication to treat NICTH, although evidence is extremely limited. The only case reported in the literature is that a recurrent solitary fibrous tumor with hypoglycemia rapidly restored normoglycemia with alpelisib 300 mg daily [14]. As a new generation of chemotherapy for HER-2 negative breast cancer, alpelisib acts by inhibiting phosphatidylinositol 3 (PI3) kinase, thus attenuating the AKT phosphorylation and glucose uptake. That is, alpelisib prevents the pro-IGF-2 stimulated insulin-IGF-I signaling [14]. Hyperglycemia is the most common adverse event of alpelisib, affecting up to 65% of patients [15]. Another approach to target this pathway is a human monoclonal antibody, RZ358, which acts as a negative allosteric modulator of insulin receptors. This investigational medication has not been approved by the FDA and was reported successfully in a single case of malignant insulinoma [16]. An approval for alpelisib was applied for compassionate use in our patient. She was unable to undergo a trial of alpelisib due to clinical deterioration and passed away.

In conclusion, our case of a patient with ACC-induced NICTH not only provided data on NICTH diagnosis and treatments but also emphasized the limited approaches to manage this severe hypoglycemic crisis. It is especially challenging as the underlying malignancy was not responding to anticancer therapies, and the comorbidities restricted the rate of dextrose infusion. Future studies are warranted to establish the efficacy of new targeted therapy that specifically modulates the IGF-2–activated insulin pathways.

## Learning Points

- NICTH is a rare but morbid complication of cancers, including ACC.
- Aggressive ACC-induced NICTH is refractory to numerous therapeutic interventions, including chemotherapy and targeted therapies.
- Existing medical therapies for NICTH are limited, which emphasizes the need for novel therapeutic strategies to be investigated in the future.

## Contributors

Y.T., O.H., S.A., L.J., J.A., and A.C. were involved in the diagnosis and management of the patient. L.J. assisted with ACTH staining of samples and pathology readings. All authors edited, reviewed, and approved the final draft.

## Data Availability

Data sharing is not applicable to this article as no datasets were generated or analyzed during the current study.

## Abbreviations

ACC: adrenal cortical carcinoma
ACTH: adrenocorticotropic hormone
CT: computed tomography
GH: growth hormone
IGF-2: insulin-like growth factor 2
IV: intravenous
NICTH: non-islet cell tumor hypoglycemia
